# Description of non-polio enteroviruses identified in two national surveillance programmes in South Africa

**DOI:** 10.4102/sajid.v35i1.196

**Published:** 2020-12-15

**Authors:** Wayne Howard, Dana Savulescu, Leigh Berrie, Adrian J. Puren

**Affiliations:** 1 National Institute for Communicable Diseases, Johannesburg, South Africa; 2 Faculty of Health, University of Witwatersrand, Johannesburg, South Africa; 3 National Priority Programmes, National Health Laboratory Services, Johannesburg, South Africa

**Keywords:** human enteroviruses, meningitis, NICD, stool specimens, disease-associated serotypes, South Africa

## Abstract

**Background:**

Human enteroviruses (EV) consist of 106 serotypes and four species: EV-A, EV-B, EV-C and EV-D. Enteroviruses cause clinical symptoms varying from severe to mild. Knowledge of EV burden in South Africa is limited, and as non-polio EV are important causes of acute flaccid paralysis (AFP) and meningitis, information on the circulating serotypes is vital.

**Methods:**

Between 2010 and 2012, a total of 832 stool and viral isolate specimens were obtained from two national surveillance programmes at the National Institute for Communicable Diseases: the Rotavirus Sentinel Surveillance Programme (RSSP) and the AFP surveillance programme. Real-time polymerase chain reaction and Sanger sequencing were performed to detect and serotype EV.

**Results:**

Non-polio EV were detected in 446 specimens, of which 308 were sequenced. Stool specimens yielded a greater variety of serotypes than viral cultures. EV-B viruses were predominant (58.44%), whilst EV-C viruses were detected in 31% of the specimens tested. South African prevalence for these viruses was higher than other countries, such as France with less than 2%, and Spain and the United States with less than 10%. The most common serotype detected was Enterovirus 99 (EV-C, 8.63%), which has not been reported in other regions.

**Conclusion:**

Direct sequencing from stool specimens yields a broader, more comprehensive description of EV infections compared to sequencing from viral cultures. Disease-associated serotypes were detected, but only in small numbers. This study provides a baseline for EV strain circulation; however, surveillance needs to be expanded to improve EV knowledge in South Africa.

## Introduction

Human enteroviruses (EV), a part of the *Picornaviridae* family in the Enterovirus genus,^1^ are divided into four species: EV-A, EV-B, EV-C and EV-D.^2^ Although EV infections occur in early childhood, they may also occur in later years because of the high number of EV serotypes.^3^ Despite most infections being asymptomatic, large numbers of symptomatic infections are estimated to occur every year, contributing to morbidity and mortality.^3^ Most EV infections are mild, causing headaches, rhinitis and rash; however, some infections can lead to serious diseases such as cardio myelitis, flaccid paralysis and diarrhoea, particularly in infants^4^ and the immunocompromised.^5^ Enteroviruses are the leading cause of viral aseptic meningitis,^6^ and are also implicated in a wide range of acute and chronic infections ranging from non-febrile disease, conjunctivitis and upper respiratory infections, to hand-foot-and-mouth disease.^6^

Enterovirus incidence is difficult to determine as transmission can be endemic, sporadic or epidemic. Additionally, many infections are subclinical.^2^ Some EV cause multiple syndromes that may develop into serious complications from asymptomatic or subclinical infections.^6^ Large populations often have multiple serotypes circulating concurrently. Outbreaks can occur with varying degrees of severity and are generally characterised by serotype, time, location and disease.^7,8^

Most studies on EV in South Africa have focused on polioviruses (PV) or outbreaks of non-polio EV.^9,10,11,12,13^ Polioviruses have caused significant outbreaks of acute flaccid paralysis (AFP) in the past and have been the focus of vaccine programmes, with the aim of eradication, since 1988.^14^ Acute flaccid paralysis is caused by PV infection of spine nerve cells, leading to irreversible damage that results in permanent paralysis or death.^5^ Studies investigating EV outbreaks in South Africa have been short term, looking at one particular virus-associated disease.^12,13^ An earlier retrospective study on a meningitis outbreak described the prevalence of non-polio EV in Cape Town in 1993.^11^ A recent study from South Africa examined the prevalence of EV in respiratory disease patients from 2009 to 2014, using respiratory swabs and lavages.^9,10^ These studies give an incomplete view on EV prevalence and have limited use in outbreak control or disease surveillance. Typing EV has evolved with the development of molecular analysis technology. Polymerase chain reaction (PCR) and genetic sequencing are now used to genotype EV into the conventionally assigned species and serotypes.^15^

Detection and elimination of PV has been achieved through the AFP surveillance network, which surveys all AFP cases detected, including those where polio is not the cause.^14^ Poliovirus was eliminated from South Africa in 1989 and thus the investigation of AFP cases remains essential because other non-polio EV may be causative organisms.^16,17^ The circulation and changes in predominance of EV serotypes are complex, and surveillance programmes may aid in tracking and identifying EV serotypes.^18^ Two routine surveillance systems in South Africa – the AFP surveillance network and the Rotavirus Sentinel Surveillance Programme (RSSP) – provide potential specimens to investigate circulating EV. Specimens from these two programmes may enable detection of EV potentially causing AFP, as well as those causing diarrhoea or gastroenteritis.

The AFP surveillance network collects stool specimens from children under the age of 15 with AFP, or adults with AFP where polio is suspected. The RSSP collects stool specimens from patients under the age of 5 admitted to hospital for diarrhoea to determine the effectiveness of the rotavirus vaccine introduced in 2009.^19^

Whilst this study sought to obtain detailed information on EV circulation in South Africa, the specimen type was limited. Cerebrospinal fluid (CSF), conjunctivitis swabs, rash vesicle fluid and respiratory and stool specimens should be surveyed to establish a complete picture of EV circulation and disease burden.^20^

This study aimed to determine the epidemiology of non-polio EV circulating in South Africa from 2010 to 2012. We investigated any serotype-disease association in stool specimens obtained from AFP suspected infections, which may give an indication of EV associated with neurological infections; and from patients with diarrhoea, elucidating EV involvement in enteric diseases and the expansion of EV surveillance.

## Materials and methods

### Specimen sources

Eight hundred and thirty-two stool and viral isolate specimens, collected between January 2010 and December 2012, were sourced from the AFP surveillance programme and the RSSP at the National Institute for Communicable Diseases (NICD), Johannesburg, South Africa.

Specimens from the AFP surveillance programme were selected by obtaining all the positive non-polio EV viral isolates determined through EV-like cytopathic effect (CPE).^21^ One stool specimen that showed no CPE on cell culture from each of the nine provinces in South Africa each month was also included for direct detection. A total of 175 non-polio EV-positive cultures were collected between 2010 and 2012, and a further 95 culture-negative stools were obtained from January to December 2012. Specimens were discarded on an annual basis and thus no raw stools were available for 2010 and 2011.

The RSSP supplied 562 stool specimens from four provinces – Gauteng, Kwa-Zulu Natal, Western Cape and Mpumalanga – covering a mixture of rural, peri-urban and urban populations.^19^ The hypothesised percentage frequency was based on the number of non-polio EV detected in the AFP surveillance network for South African patients per year. We selected the first four specimens arriving at the NICD from each site per month for the years 2010–2012 calculated by Equation 1:

$$n=\left[{\text{Z}}^{2}*\text{p}*(1-\text{p})\right]/{\text{c}}^{2}$$ [Eqn 1]

- *n* = sample size
- *p* = hypothesised percentage frequency of outcome (15%), confidence level of 95%
- *c* = 0.05
- *Z* = 1.96

The Western Cape started collecting specimens in May 2010, resulting in a lower total specimen number for 2010 (175 specimens) from that site.

### Specimen preparation

Viral Ribonucleic Acid (RNA) extractions were conducted on culture samples and stool samples using the automated Maxwell 16 system (Promega, Madison, Wisconsin, United States), or manually using the Qiagen Qiamp Viral Mini Kit (Qiagen, Venlo, Netherlands). For stool samples, both manual and automated extractions were preceded by stool dilution in stool transport and recovery (STAR) buffer (Roche, Mannheim, Germany), to ensure adequate removal of (PCR) inhibitors. The treated stool specimens were centrifuged at 1500 g for 1 min at room temperature to sediment the solids, with the supernatant aliquoted. Specimens that failed to yield a useable nucleotide sequence were processed manually and re-sequenced.

### Polymerase chain reaction and sequencing

The real-time PCR protocol from Nijhuis et al.^22^ was used to screen specimens for the presence of EV, followed by amplification and sequencing of EV-positive specimens using a semi-nested assay and degenerate PCR primers, sequencing primers and protocols designed by Nix et al.^23^

Sanger sequencing was conducted as per the BigDye Terminator (version 3.1) Cycle Sequencing Kit (Life Technologies, Carlsbad, California, United States) and analysed on the ABI 3130 genetic analyser (Life Technologies, Carlsbad, California, United States).

Specimens were serotyped using Oberste’s criteria for EV typing, that is, greater than 75% nucleotide sequence homology to the published sequences.^15^ The National Centre for Biotechnology Information (NCBI) database was utilised to compare the EV sequences obtained in the study using the BLAST (Basic Local Alignment Search Tool) function.

### Statistics

The gender prevalence and median age of the selected cases were calculated from surveillance data available, with the interquartile range determined by subtracting the lower quartile from the upper quartile. One-way Analysis of Variance (ANOVA) and *T*-tests were done on GraphPad Prism (University of Leicester, United Kingdom). A *p* value of 0.05 and lower was considered statistically significant.

### Ethical consideration

Ethics approval was obtained from the University of Witwatersrand Ethics Committee (M120467, M119034 and M111145).

## Results

The EV PCR screen was conducted on 832 specimens with 446 (53.61%) specimens reported positive for EV. Male patients constituted 55.51% (246/446) of the EV-positive cases; most specimens came from children under the age of 5, with a median age of 1 year and interquartile range of 1.48. Patients under the age of 1 made up 49.33% (220/446) of the positive specimens, 41.26% (184/446) were between the ages of 1 and 5 years and the remainder (7.62%; 34/446) over the age of 5 (Table 1). The unaccounted for ages (1.79% (8/446) and gender (2.47% 11/446) are unknown.

**TABLE 1:** Gender and age of patients with enterovirus-positive specimens.

Variable	%
**Gender**	
Male	55.16
Female	42.38
**Patients’ age†**	
< 1 year old	49.33
> 1 year and < 5 years old	41.26
> 5 years old	7.62

† , 90.59% under 5 years old.

Sixty-three serotypes were detected from three species groups, EV-A, EV-B and EV-C. No EV-D serotypes were identified. EV-A, EV-B and EV-C were detected in 10.7% (33/308), 58.4% (180/308) and 30.8% (95/308) of specimens, respectively. In EV-A, there were 12 serotypes identified, with CVA5 most frequently detected (6/32 detections, 18.2%). Thirty-seven EV-B serotypes were detected with the most common serotype being CVB3 (in 15/180 [8.3%] specimens), followed by Ec6 (14/180 specimens, 7.8%). EV-C had 14 serotypes, where EV99 was detected in 27 of 95 specimens (28.4%) and CVA24 in 16 of 95 specimens (16.8%) (Table 2).

**TABLE 2:** Total number of serotypes detected.

Species	Serotype	Overall detections	Individual percentage of total detections
EV-A†	CA2	4	1.30
	CA4	1	0.32
	CA5	6	1.95
	CA6	3	0.97
	CA7	3	0.97
	CA8	1	0.32
	CA10	4	1.30
	CA14	2	0.65
	CA16	3	0.97
	EV71	2	0.65
	EV76	1	0.32
	EV114	3	0.97
EV-B‡	CB1	5	1.62
	CB2	4	1.30
	CB3	15	4.87
	CB4	4	1.30
	CB5	8	2.60
	CB6	1	0.32
	CA9	3	0.97
	Ec1	3	0.97
	Ec2	1	0.32
	Ec3	8	2.60
	Ec4	2	0.65
	Ec5	1	0.32
	Ec6	14	4.55
	Ec7	6	1.95
	Ec9	7	2.27
	Ec11	10	3.25
	Ec12	5	1.62
	Ec13	11	3.57
	Ec14	7	2.27
	Ec15	4	1.30
	Ec16	3	0.97
	Ec17	1	0.32
	Ec18	1	0.32
	Ec19	9	2.92
	Ec20	6	1.95
	Ec21	7	2.27
	Ec24	6	1.95
	Ec25	7	2.27
	Ec27	4	1.30
	Ec29	4	1.30
	Ec30	5	1.62
	Ec31	1	0.32
	Ec32	1	0.32
	EV75	2	0.65
	EV77	1	0.32
	EV80	2	0.65
	EV88	1	0.32
EV-C§	CA1	2	0.65
	CA11	4	1.30
	CA13	11	3.57
	CA17	4	1.30
	CA19	3	0.97
	CA20	2	0.65
	CA21	2	0.65
	CA22	4	1.30
	CA24	16	5.19
	EV99	27	8.77
	EV102	1	0.32
	PV1-SABIN	2	0.65
	PV2-SABIN	5	1.62
	PV3-SABIN	12	3.90
**Total**	-	308	-

† , Species group EV-A (%) = 10.71;

‡ , Species group EV-B (%) = 58.44;

§ , Species group EV-C (%) = 30.84.

The AFP surveillance programme yielded 168 of the 270 (62.22%) EV-positive cases from the culture and stool specimens, of which 147 (87.5%, *n* = 168) were serotyped. The species groups detected included EV-A (7.5%; 11/147), EV-B (79.6%; 117/147) and EV-C (12.9%; 19/147). The most common serotype was CVB3, followed by Ec6. Enterovirus 99 was detected in seven specimens: three from viral culture positive cases, and four from AFP stool specimens.

From the RSSP subset, 49.11% (276/562) were positive for EV. Serotypes were identified from 58.3% (161/276) of stools including species group EV-A (13.7%; 22/161), EV-B (39.1%; 63/161) and EV-C (47.2%; 76/161). Enterovirus 99 was the most frequent serotype detected (12.4%; 20/161), followed by CVA24 (8.7%; 14/161).

**FIGURE 1:**
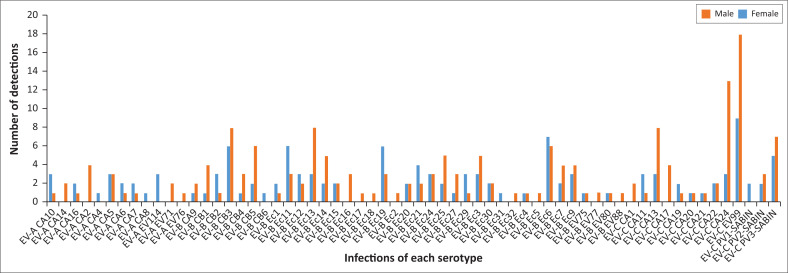
Infections of each serotype in males and females.

Although EV-B was the predominant group detected overall, EV99 from the EV-C group was the serotype most frequently detected overall (8.8%; 27/308), followed in decreasing frequency by CVA24 (5.2%, EV-C; 16/308), CVB3 (4.87%, EV-B; 15/308) and Ec6 (4.55%, EV-B; 14/308). The serotypes detected from EV-A, EV-B and EV-C groups were heterogeneous and there was no dominant EV-A and EV-B serotype. EV-A and EV-B serotypes did not show an infection bias towards gender, although EV-C infected more males (61/94 detections, 64.89%) than females (33/94 detections, 35.11%) (Figure 1). This was not statistically significant (*p*-value = 1.00, confidence interval [CI]: 95%).

Specimen type influenced the groups and serotypes identified. Many of the AFP programme specimens yielded EV-B (90.4%; 113/125) strains. Two serotypes were most frequently detected: CVB3 in 13 specimens and Ec6 in 12 specimens. The stool specimens yielded mainly EV-C serotypes: 63.6% (14/22) from the culture-negative, AFP stool specimens; and 47.2% (76/161) from the RSSP specimens. Enterovirus 99 was the most frequently detected serotype in the combined sets of stool specimens. Enteroviruses CVA1, 5, 8, 9, 10, 11, 13, 15, 17, 24, and Ec15, were serotypes detected in AFP stool specimens, but not from cell culture.

**FIGURE 2:**
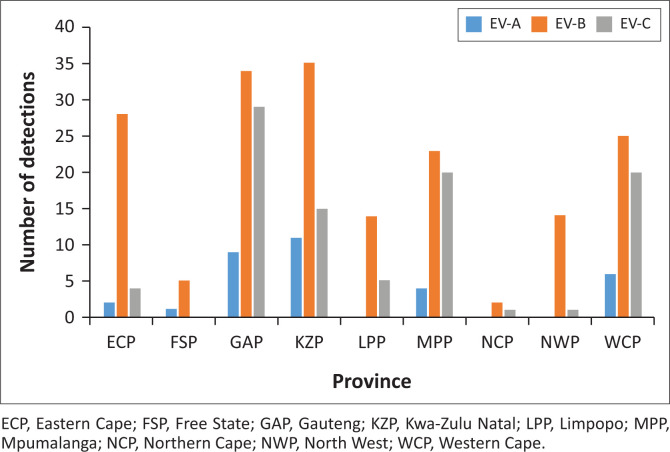
Distribution of EV-A, EV-B and EV-C per province in South Africa.

The distribution of EV serotypes was varied and widespread across the country and no distinct distribution pattern in the different provinces was observed. Most specimens were collected from the provinces that included the RSSP collection sites as well as from the AFP surveillance programme: Western Cape, Gauteng, Kwa-Zulu Natal and Mpumalanga (Figure 2).

**FIGURE 3:**
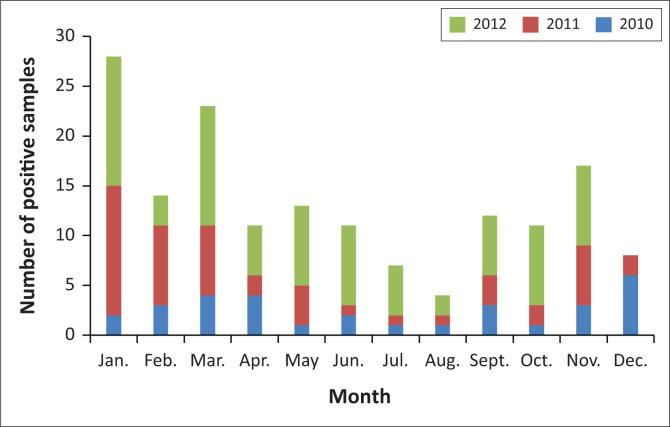
Total number of positive specimens in the acute flaccid paralysis surveillance programme by month, for 2010–2012.

There was no clear seasonality in the distribution of the positive EV specimens detected in the RSSP. In the case of the AFP surveillance, seasonality was observed with January to March showing a peak in infections (Figure 3), although this was not statistically significant (*p*-value = 0.4433; CI: 95%).

## Discussion

No predominant EV serotype was detected, and a wide distribution of serotypes across EV-A, EV-B and EV-C was observed. Serotype distribution followed for the most part gender and age patterns seen historically across the world.^2,3,5,6^ Some known disease-associated serotypes were detected but were not more prevalent than other serotypes.

The AFP surveillance programme obtained specimens from all nine provinces, which allowed for description of a countrywide EV distribution over a 3-year period. The use of this programme was advantageous as the specimens originated from all provinces and most districts in the country, and the infrastructure for specimen collection and transport had already been established.

The RSSP^19^ sites covered different regions in South Africa and overlapped areas covered by the AFP surveillance programme. The sites were in the Gauteng, Western Cape, Kwa-Zulu Natal and Mpumalanga provinces.^24^ Using the national surveillance systems in place ensured good population coverage and direct stool specimen screening, without a virus isolation intermediate step. This allowed for detection of serotypes that are impossible or difficult to grow in cell cultures.

The pattern of EV distribution seen in the cell culture specimens was consistent with other studies worldwide,^25,26,27,28,29,30,31^ with EV-B being the predominant group and EV-D being the least prevalent group. However, the stool specimens screened in our study yielded additional EV-C viruses (47.20% in the RSSP specimens and 63.64% in the AFP stool specimens). Our data show that using only cell culture for EV identification may limit and bias the results towards isolating EV-B viruses.

Although EV-B was the most prevalent species detected, EV99 (EV-C) was the most prevalent serotype detected over 3 years – 27 out of 308 specimens typed (8.63%). Within each year, EV99 was the most prevalent serotype found along with Ec6 (9 viruses, 2010), CVB5 and Ec14 (6 viruses each, 2011) and Ec13 (10 viruses, 2012). This finding was unusual, as EV99 has not been previously detected as a common serotype in other studies in China and Finland.^32,33^

Despite many serotypes detected in our study being associated with disease, these serotypes did not contribute significantly to the total number of viruses detected. Strain EV71 (isolated from the AFP culture positive specimens) has been associated with aseptic meningitis,^34,35^ and although its presence in patients presenting with neurological symptoms is expected, only two cases were detected over the three surveillance period. The more recently classified EV, namely, EV80, EV88, EV102 and EV114, were also detected in this study. These viruses are rarely detected and/or newly discovered and have no clear disease association.

The distribution of the serotypes across the country did not show any distinct pattern, although many more types were detected in Gauteng, Mpumalanga, Kwa-Zulu Natal and Western Cape. This is likely because of the larger numbers of specimens obtained from these provinces, as well as their mixed populations. Further studies, with specimens collected more evenly between the nine provinces, will be required in order to confirm this. Ideally, a surveillance programme tailored to detect EV symptoms including hand-foot-and-mouth disease, aseptic meningitis, myocarditis and respiratory disease would be required for improved detection.^9,10,18,36^

Serotype distribution varies over time within a geographical location,^37^ as well as over large distances, such as between continents. Enterovirus Ec30 is the predominant serotype in Europe,^38^ whilst EV71 is the predominant serotype worldwide.^39^ In our study, EV99 was found to be the most common serotype in South Africa. Other studies^32,33^ have not definitively linked EV99 to a disease, and with the virus still relatively unknown, further investigations are required to discover any clinical relevance. The higher levels of EV99 detected compared to other countries may be because of lack of serotyping studies, as well as the use of stool specimens in this study instead of viral isolates.^7,8,40,41^ Enterovirus 99 does not grow in cell cultures as readily as viruses from EV-B. Detection of EV directly from stool specimens allows for a more accurate distribution of EV within a population. This genotyping method is faster than virus isolation and so results from an outbreak can be utilised with epidemiological information to prevent further transmission.^23^

Hellferscee and colleagues^9,10^ published two studies that showed EV strains in respiratory patients from 2009 to 2014 in South Africa, with a wide variety of serotypes detected. Their results correlate with our findings, although we did not detect EV68, more common in respiratory specimens. As EV68 has been shown to be associated with respiratory disease, this is not unexpected.^42,43^ All serotypes detected by Hellferscee *et al.*, except CVA3 and EV68, were detected in our study.

This study provides a baseline for EV strain circulation and epidemiology in South Africa. With polio on the brink of eradication, other causes of AFP need to be investigated. Enteroviruses are a potential cause of these symptoms^39^, and this study supplies a baseline for determining which EV are circulating in the South African population. Whilst EV71, a meningitis-associated virus, and various other echoviruses were detected in this study, routine and outbreak surveillance will determine the clinical importance of these serotypes in the South African population. Inclusion of a surveillance programme would assist in detecting EV outbreaks, and utilisation of various specimen types would ensure that EV that replicate in different organs (e.g. EV68 found in respiratory lavages and/or swabs) were not missed.

## Limitations

Limitations of this study include no control group of healthy individuals for comparison, although a study in the Philippines shows no difference between diseased and asymptomatic groups.^44^ The differences in serotype distribution between the surveillance programmes may have been because of the specimen type. The less severe symptom types associated with EV are not currently covered by any surveillance group in South Africa.

The current surveillance programmes in South Africa used to collect specimens target specific age groups (mostly children under 5 or 15) and are passive systems; consequently, specimen collection is only triggered when syndromes are detected. The AFP surveillance programme met all surveillance targets for the number of specimens collected in all provinces except the Northern Cape. This may underrepresent the number of viruses typed in this province.

Only one type of specimen, namely faeces, was collected from the surveillance programmes in this study, making it more difficult to obtain results (PCR inhibitors are difficult to remove and there is a risk of mixed EV infections).

Enterovirus genotyping has become more complex with the discovery of more serotypes. A small fragment of the VP1 gene was used for typing the EV, which was sufficient for basic typing differentiation, but more in-depth genetic analysis is required for a comprehensive description of EV in South Africa.

## Conclusion

The epidemiology of EV in South Africa showed a general concordance with other studies^16,25,29^, and the study provided a baseline of circulating EV strains. In South Africa, various serotypes were shown to co-circulate, although EV99 was the most common virus throughout the 2010–2012 period. Strains CVA24 and EV99 accounted for 14% of all viruses detected over the 3 years and the predominance may be explained by the natural continental differences in serotype circulation.

Specimen type influences the ability to detect different serotypes, and disease presentation affects the serotypes observed. Future surveillance may assist in determining how serotype affects disease burden. Unlike cell culture, an assay that will detect EV directly from the specimen may give a more comprehensive idea of EV strain circulation and epidemiology.

A dedicated EV surveillance programme would provide a more accurate idea of the EV disease burden on symptoms such as AFP, meningitis and encephalitis. This would be useful for outbreak detection and virological investigation.^36^ The development of a new vaccine to lessen the disease burden of serotypes with the association of serious effects on patients may be a by-product of the knowledge gained from EV surveillance, as predominant serotypes can be investigated as vaccine candidates.
